# Lassa and Mopeia viruses produce different RIG-I-activating RNA in the absence of a functional viral exoribonuclease domain

**DOI:** 10.1128/jvi.02110-25

**Published:** 2026-05-11

**Authors:** Kodie Noy, Rachel Legendre, Séverine Croze, Valérie Najburg, Romane Fernandez, Julie Lucifora, Joel Lachuer, Sylvain Baize, Anastassia V. Komarova, Mathieu Mateo

**Affiliations:** 1 Biology of Viral Emerging Infections Unit, Institut Pasteur, Université Paris Cité 555089, Paris, France; 2 Centre International de Recherche en Infectiologie (CIRI), Université de Lyon, Inserm U1111, Université Claude Bernard Lyon 1, CNRS UMR5308, ENS de Lyon 133614, Lyon, France; 3 Bioinformatics and Biostatistics Hub, Institut Pasteur, Université Paris Cité 555089, Paris, France; 4 ProfileXpert, Faculté de Pharmacie de Lyon, Université de Claude Bernard Lyon 1, UMS 3453 CNRS – US7 INSERM 70614 , Lyon, France; 5 Institut Pasteur-Oncovita Joint Laboratory, Université Paris Cité, Institut Pasteur 555089, Paris, France; 6 Interactomics, RNA and Immunity Laboratory, Université Paris Cité, Institut Pasteur 555089, Paris, France; Emory University School of Medicine, Atlanta, Georgia, USA

**Keywords:** viral RNA, interferon response, RIG-I-like receptors, arenaviruses

## Abstract

All viruses of the genus *Mammarenavirus* possess an exoribonuclease (ExoN) domain in their nucleoproteins (NP). Through this domain, the NP efficiently prevents the activation of the interferon (IFN) response, presumably by degrading double-stranded RNA (dsRNA) produced during the viral replication. While the importance of this ExoN activity regarding *Mammarenavirus* virulence is well established, little is known about the dsRNA molecules that are targeted by NP for degradation, and the contribution of cellular sensors activated by these molecules has yet to be described. Here, we addressed these questions using recombinant viruses with abrogated ExoN domains that are no longer able to control the IFN response. We infected RIG-I, MDA5, and MAVS deficient cells and demonstrated that ExoN mutants activate the interferon response through RIG-I, but not MDA5. We then purified RIG-I-associated RNA from infected cells and confirmed its immunostimulatory activity in transfected cells. We sequenced the RIG-I-associated RNA and identified different enriched sequences in the Mopeia virus (MOPV) or Lassa virus (LASV) RNA. RIG-I activating sequences corresponded to the extremities of the 5′ ends and intergenic regions of the MOPV genome and to a glycoprotein precursor complex (GPC) sequence in the LASV genome. We produced corresponding synthetic RNA molecules and confirmed their RIG-I-dependent activation of the IFN response. These results underline the central role of the ExoN domain in MOPV and LASV NP for immune escape and identify new virus-derived RNA molecules with high immunostimulatory properties.

**IMPORTANCE:**

Arenaviruses prevent the activation of the interferon response due to the exonuclease activity of their nucleoprotein, suggesting that infection leads to the production of immunostimulatory RNA molecules. However, neither the exact nature of the immune RNA sensors nor the identity of the RNA activating these sensors is clearly identified. By taking advantage of recombinant MOPV and LASV deficient for their exonuclease activity and that are strong activators of the interferon response, we have identified RIG-I as the major sensor of arenaviruses in infected cells. We also identified the RNA molecules recognized by RIG-I upon infection, and we highlighted differences between MOPV and LASV viruses. Our results represent critical information regarding the factors of immunogenicity and pathogenicity of Old-World arenaviruses and can help us explain key differences between pathogenic and non-pathogenic arenaviruses.

## INTRODUCTION

In the recently regrouped *Hareavirales* order, the family *Arenaviridae* comprises *Mammarenavirus*, *Reptarenavirus*, *Hartmanivirus, Antennavirus,* and the current but potentially temporary genus *Innmovirus*. Among the genus *Mammarenavirus* are distinguished New World arenaviruses (NWA) circulating in the Americas and Old-World arenaviruses (OWA) circulating in the rest of the world. Several human pathogens are found among the Mammarenavirus genus, including NWA such as Junín (JUNV), Chapare, Gunanarito, Machupo, and Sabiá viruses, and the OWA Lassa (LASV) and Lujo viruses. Lymphocytic Choriomeningitis virus (LCMV), the prototypic OWA, is the only known mammarenavirus circulating globally. LASV is an OWA of major public health concern, and Lassa fever is responsible for thousands of deaths each year in West Africa. LASV has therefore been proposed within the list of top priority pathogens by the World Health Organization. The discovery of new circulating *Mammarenavirus* species also raises concerns about possible novel emergence events in the human population. However, most *Mammarenavirus* species may not be pathogenic for humans, such as the African Mopeia virus (MOPV) that has never been associated with any human disease, despite being genetically close to its pathogenic counterpart LASV (1).

*Mammarenavirus* species differ in their pathogenic potential, but they all share the same genomic organization. The *Mammarenavirus* genome is bi-segmented, and the two genes on each segment are transcribed using an ambisense coding strategy. In this way, the gene located on the 3′ end of each segment is directly transcribed from genomic RNA; however, anti-genomic RNA is required for the transcription of the remaining gene close to the 5′ end. The “short” segment (S) encodes, from 3′ to 5′, the nucleoprotein (NP) and the glycoprotein precursor complex (GPC, which is post-translationally cleaved into GP1 and GP2). The “long” segment (L) encodes the RNA-dependent RNA polymerase L (L) and the Z matrix protein (2). Genes present on a single segment are separated by an intergenic region (IGR) proposed to form a stable hairpin that serves as a transcription termination signal.

Among the viral proteins, NP is a major virulence factor. NP encapsidates the viral genomes and antigenomes and serves as a cofactor for the L polymerase. In addition, NP prevents the activation of the innate antiviral response. The interferon (IFN) antagonist function of the *Mammarenavirus* NP was first discovered by Martinez-Sobrido et al. in 2007 when they demonstrated that the LCMV NP could counteract the induction of IFN by viral or synthetic RNA (3). They then demonstrated that this IFN-antagonist was conserved among *Mammarenavirus* species and identified several amino acids in the C-terminal domain of NP participating in this function (4). Shortly after, the crystal structure of Lassa NP was solved, highlighting the presence of an exoribonuclease (ExoN) domain responsible for the inhibition of RNA-driven activation of the interferon response (5, 6). This ExoN domain is a DEDDh domain with 3′→5′ ExoN activity that is similar to the ones observed in interferon-stimulated gene 20 (ISG20) and three prime repair exonuclease 1 (TREX1), showing specificity for double-stranded RNA (dsRNA) (6, 7). We later demonstrated that introducing mutations in the ExoN domain of a recombinant LASV abrogated its ability to control the IFN response, leading to strong attenuation in antigen-presenting cells that are believed to be the first targets of infection (8). The role of the ExoN domain in pathogenicity was also confirmed *in vivo* using a lethal model of Pichinde virus (PICV) infection (9).

The discovery of the ExoN domain strongly suggested that viral production of dsRNA during the replication of *Mammarenavirus* species could activate dsRNA sensors, resulting in the induction of an antiviral response. Toll-like receptor (TLR) 3 and the RIG-I-like receptors (RLRs; retinoic acid-inducible gene I or RIG-I, and melanoma differentiation-associated protein 5 or MDA5) are the main activators of the IFN response in response to dsRNA recognition. An earlier study of chronic and acute LCMV infection in mice demonstrated the central role of RLRs in both chronic and acute infections, while TLR3 only participated in chronic infections (10). RIG-I in particular was shown to be important for the antiviral response against JUNV (11), LASV (12), LCMV (13), and PICV (14). Regarding MDA5 in these examples, data are conflicting as its role was not assessed during JUNV and LASV *in vitro* infections, was demonstrated not to participate in the antiviral response to LCMV *in vitro* (13), but participated in the control of PICV *in vivo* (14).

RIG-I and MDA5 recognize different dsRNA molecules. RIG-I preferentially binds relatively short dsRNA, critically those harboring blunt ends and a 5′ triphosphate (5′−3P) (15–17). MDA5, on the other hand, has an affinity for long dsRNA sequences. MDA5 can bind to dsRNA as short as 50–100 base pairs, but has a preference for longer dsRNA with no end specificity during ATP hydrolysis, with multiple MDA5 helicases operating on the same sequence of RNA to trigger downstream signaling (18, 19). This signaling is facilitated in RIG-I and MDA5 through their Caspase Activation and Recruitment Domain, which allows both RIG-I and MDA5 to translocate once bound to dsRNA and interact with the Mitochondria AntiViral Signal (MAVS; also known as IPS-1, VISA, and CARDIF) (20, 21). MAVS will then activate TANK-binding kinase 1 and IκB kinase-ε, which will activate Interferon Regulatory Factor 3 (IRF3) through phosphorylation and dimerization, leading to the nuclear translocation of IRF3, where it will activate the expression of type-I IFN (IFN-I) (22).

It remains unclear how arenaviruses activate RLRs and the IFN response. The team of Dominique Garcin proposed that the non-templated G added to the 5′ extremities of mammarenavirus genomes may impair the recognition of the genome extremities by RIG-I, suggesting that the genomic panhandle may not be a potent activator of the IFN response (23). The intergenic sequence forms a stable hairpin structure, but its internal localization within the arenavirus genome does not support its role as a *bona fide* activator of RIG-I. Non-standard genomes with deleted extremities or internal sequences are also produced during LCMV infections and may participate in the induction of the antiviral response. LASV may also produce viral genomes with internal deletions in the absence of ExoN activity, supporting their participation in the induction of the antiviral response (24).

In this study, we aimed at identifying how the arenaviruses MOPV and LASV activate the RLR and the IFN response. In order to highlight this mechanism and identify its cogs, we used recombinant MOPV_ExoN_ and LASV_ExoN_ viruses that are potent activators of the IFN response (8, 25). We also took advantage of genetically modified A549 cell lines to identify the relative contribution of each RLR to the induction of the IFN response and to identify the RNA molecules activating these RLRs. We demonstrated that MOPV and LASV viruses produce different viral RNA molecules that are recognized by RIG-I to activate the IFN response. These data inform us of important differences between MOPV and LASV that could explain their difference in pathogenicity and offer new avenues for the development of treatments against arenavirus infections.

## RESULTS

### MOPV and LASV induce the IFN response through RIG-I/MAVS signaling

**Fig 1:**
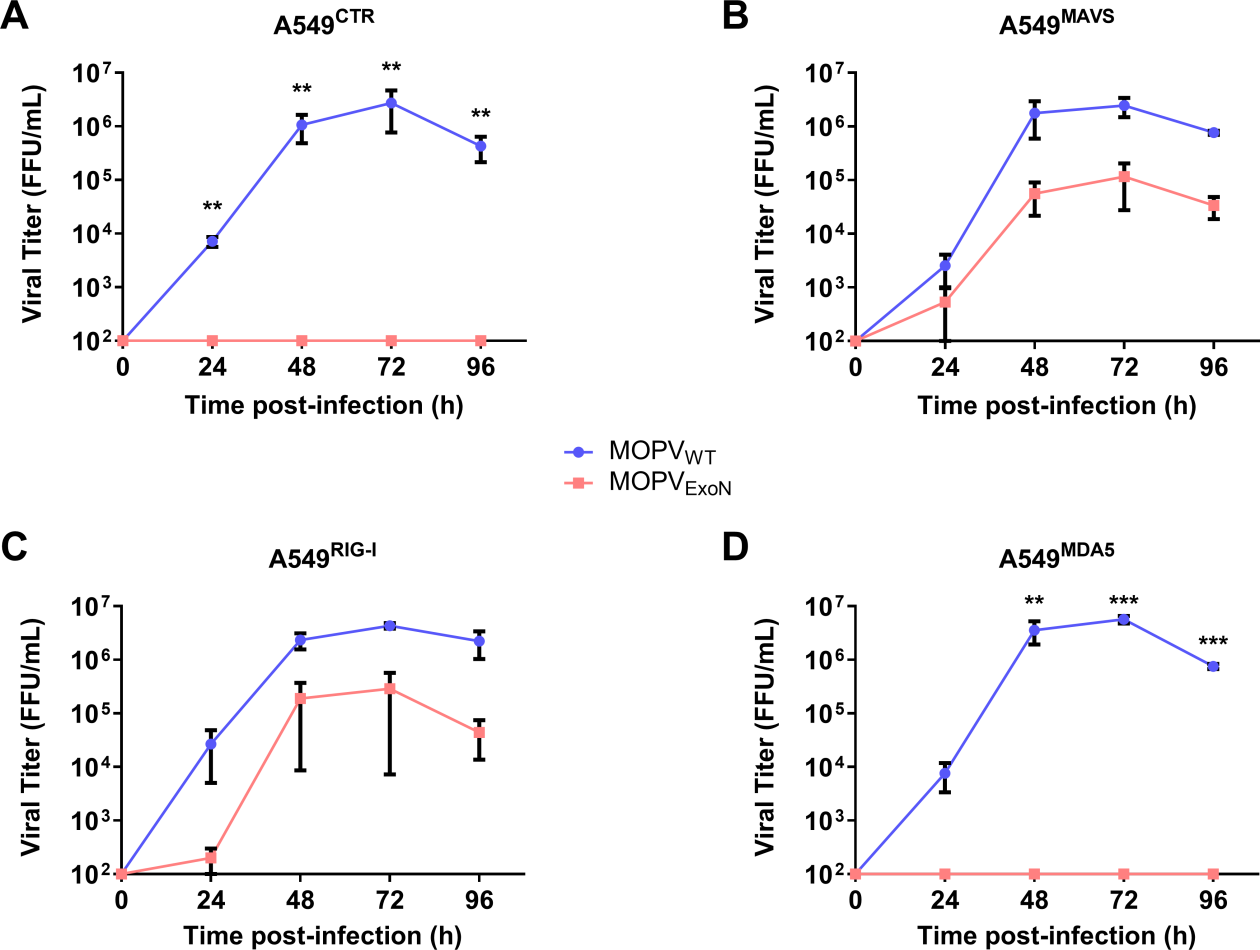
Replication of WT and ExoN MOPV in A549 cells deficient or not for MAVS, RIG-I, or MDA5. A549^CTR^ (**A**), A549^MAVS^ (**B**), A549^RIG-I^ (**C**), or A549^MDA5^ (**D**) cells were infected with recombinant MOPV_WT_ (blue circles) and MOPV_ExoN_ (red squares) at MOI 0.01. Cell supernatant was collected at 24 h intervals and analyzed by focal forming assay (FFA). Results are represented as the mean ± SEM of three biologically independent experiments, and statistical significance was determined via ANOVA (**P < 0.01, ***P < 0.001).

In order to identify the contribution of RIG-I and MDA5 in the induction of the IFN response, we decided to use genetically modified cell lines with abrogated expression of MAVS, RIG-I, or MDA5. We chose A549 cells because MOPV_ExoN_ was strongly attenuated in this cell line compared to MOPV_WT_, reproducing what we observed in antigen-producing cells (26) (Fig. S1). We infected control A549 cells (A549^CTR^), MAVS-deficient A549 cells (A549^MAVS^), RIG-I-deficient A549 cells (A549^RIG-I^), and MDA5-deficient A549 cells (A549^MDA5^) (Fig. S2) with recombinant MOPV_WT_ or MOPV_ExoN_ viruses at a multiplicity of 0.01 for 96 h and harvested supernatants every 24 h to evaluate the extent of virus replication (Fig. 1). MOPV_WT_ presented similar growth kinetics in the 4 cell lines, with peak replication at more than 10^6^ focus-forming units (FFU)/mL at 72 h post-infection. MOPV_ExoN_ did not replicate in A549^CTR^ (Fig. 1A) but was partially rescued in A549^MAVS^ (Fig. 1B) and A549^RIG-I^ (Fig. 1C) to titers slightly above 10^5^ FFU/mL. In the A549^MDA5^ cells, nevertheless, MOPV_ExoN_ did not replicate (Fig. 1D).

**Fig 2:**
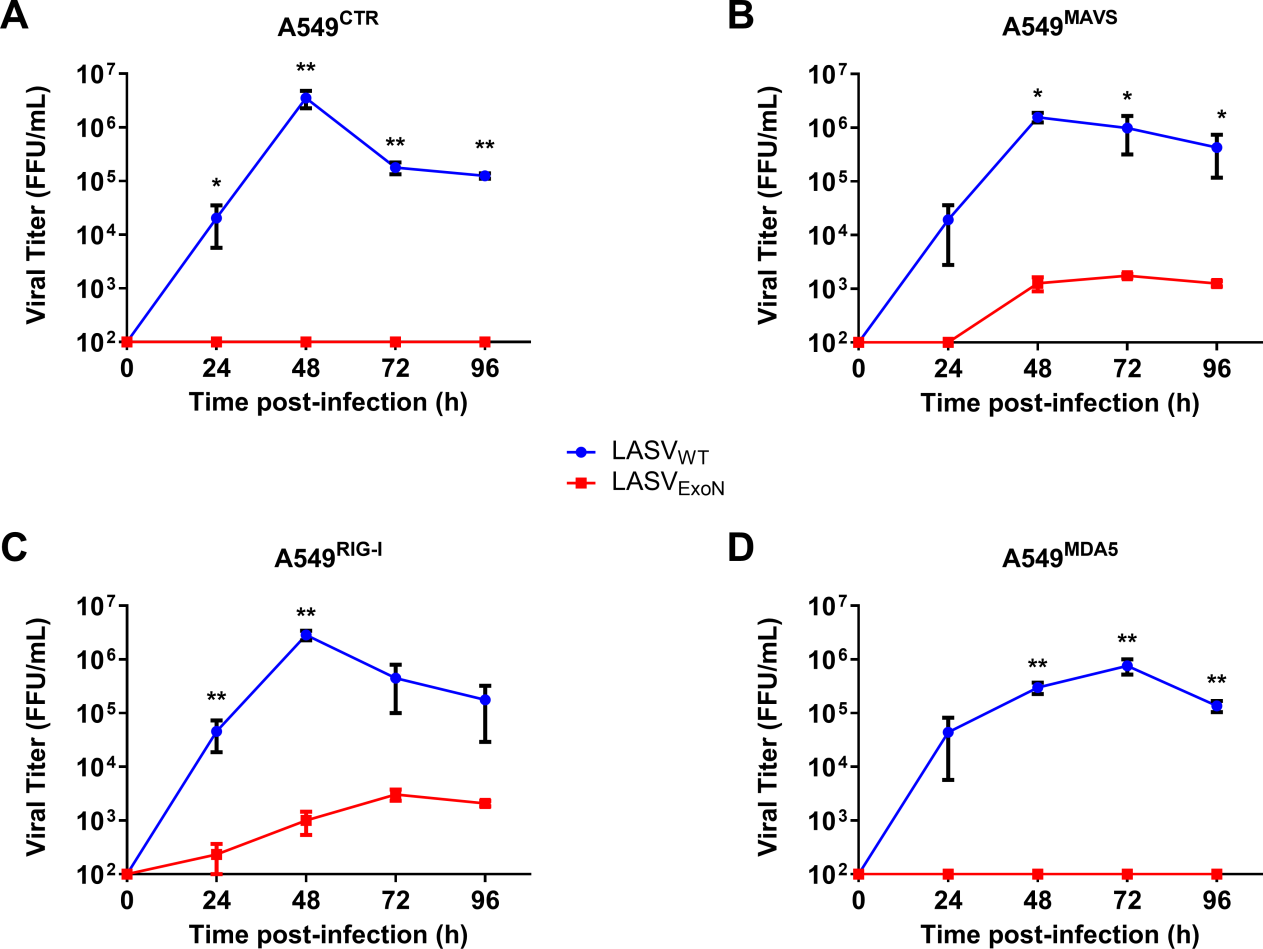
Replication of LASV_WT_ and LASV_ExoN_ LASV in A549 cells deficient or not for MAVS, RIG-I, or MDA5. A549^CTR^ (**A**), A549^MAVS^ (**B**), A549^RIG-I^ (**C**), or A549^MDA5^ (**D**) were infected with recombinant LASV_WT_ (blue circles) and LASV_ExoN_ (red circles) at MOI 0.01. Cell supernatant was collected at 24 h intervals and analyzed by FFA. Results are represented as the mean ± SEM of three biologically independent experiments, and statistical significance was determined via ANOVA (*P < 0.05, **P < 0.01).

We performed a similar experiment using recombinant LASV_WT_ and LASV_ExoN_ viruses (Fig. 2). Similar to what we observed with MOPV, LASV_WT_ replicated efficiently in the four A549 cell lines but peaked earlier at 48 h at titers above 10^6^ FFU/mL. LASV_ExoN_ replication was completely abrogated in A549^CTR^ cells (Fig. 2A), while it was only partially restored in A549^MAVS^ (Fig. 2B) and A549^RIG-I^ (Fig. 2C), but to low titers around 10^3^ FFU/mL. LASV_ExoN_ did not replicate in A549^MDA5^ cells (Fig. 2D). These results strongly suggested that RIG-I and MAVS, but not MDA5, participated in the control of MOPV_ExoN_ and LASV_ExoN_ replication in A549 cells through the induction of an antiviral response.

**Fig 3:**
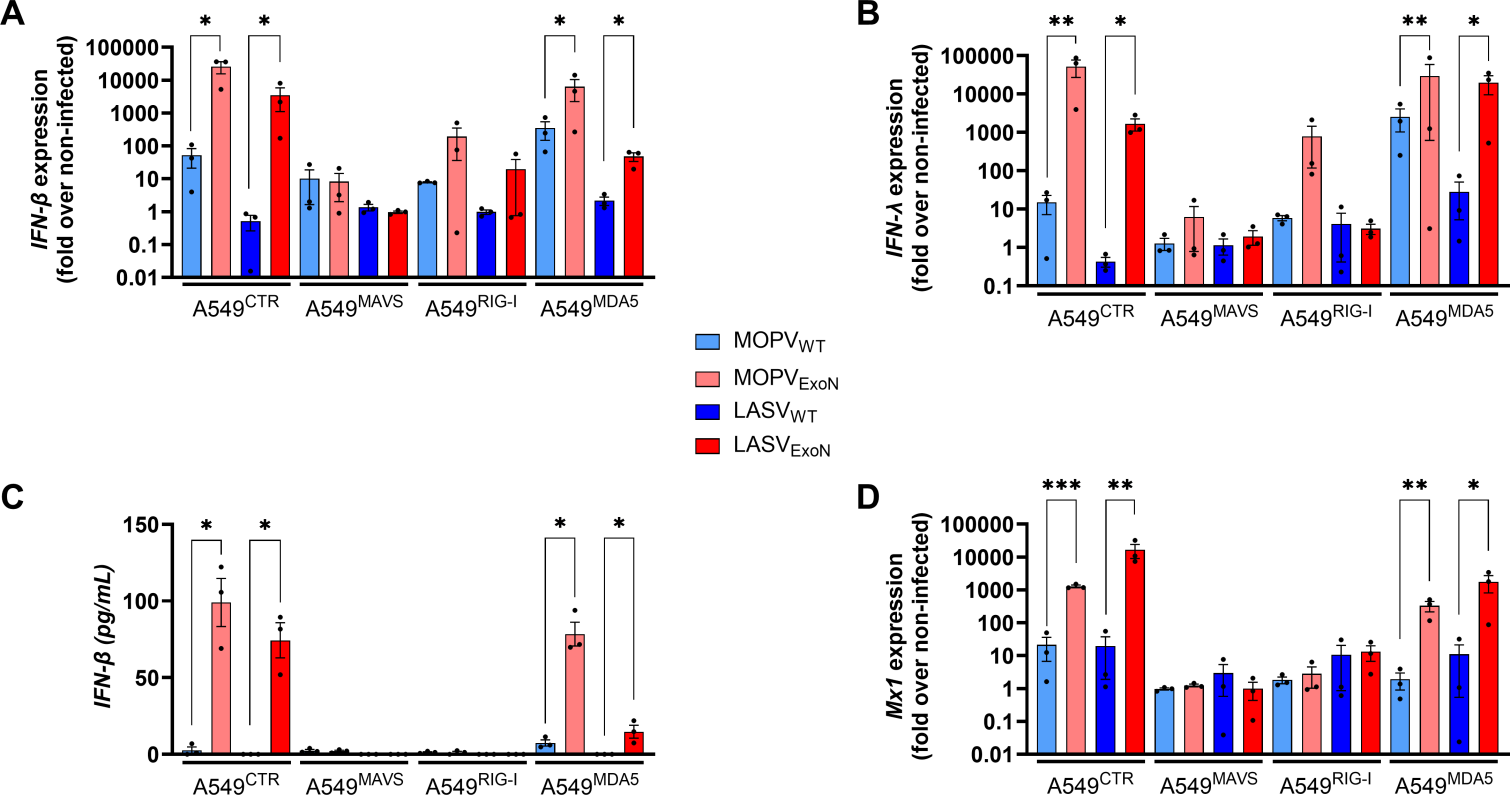
Interferon induction during WT or ExoN MOPV and LASV infection in A549 cells deficient or not for MAVS, RIG-I, or MDA5. Cells were either mock-infected or infected with MOPV_WT_ (light blue), MOPV_ExoN_ (pink), LASV_WT_ (blue), or LASV_ExoN_ (red) at an MOI of 0.01. At 48 h PI, cellular RNA was extracted and analyzed by RT-qPCR for the expression of IFN-β (**A**), IFN-λ (**B**), or Mx1 (**D**), and cell supernatant was used to measure the secretion of IFN-β from infected cells via ELISA (**C**). All figures represent the mean ± SEM of three biologically independent experiments, and significance was determined via Student's *t*-test (*P < 0.05, **P < 0.01, ***P < 0.001).

We therefore analyzed the antiviral response induced by the different recombinant viruses in the different cell lines. The expression of *IFN-β* and *IFN-λ* was significantly induced in A549^CTR^ and A549^MDA5^ cells infected with MOPV_ExoN_ or LASV_ExoN_ compared to MOPV_WT_-infected cells (Fig. 3A and B), but was not significantly induced in A549^MAVS^ and A549^RIG-I^ cells. Similarly, IFN-β secretion was only detected in the supernatants of A549^CTR^ and A549^MDA5^ cells infected with MOPV_ExoN_ or LASV_ExoN_ but not in the supernatants of cells infected with the wild-type recombinant viruses or the supernatant of A549^MAVS^ and A549^RIG-I^ cells infected with MOPV_ExoN_ or LASV_ExoN_ (Fig. 3C). In addition, MOPV_ExoN_ and LASV_ExoN_ significantly induced the expression of the ISG *Mx1* in the CTR cells and the cells lacking MDA5 expression compared to wild-type counterparts but not in the cells lacking MAVS or RIG-I expression (Fig. 3D).

We also performed a series of similar experiments in fully differentiated HepaRG cells (dHepaRG), which are a surrogate of primary hepatocytes regarding innate immune responses (27, 28) (Fig. S3). No significant change in replication was observed in MOPV_WT_ or LASV_WT_ viral replication in the absence of IRF-3, RIG-I, or MDA5 (Fig. S3A and B). MOPV_ExoN_ and LASV_ExoN_ were not completely attenuated in dHepaRG cells, with distinct profiles of the replication of these viruses between dHepaRG cells lacking IRF-3, RIG-I, or MDA5; however, no significant difference was observed at each time point. In the dHepaRG cells, we confirmed that MOPV_WT_ and LASV_WT_ did not induce any IFN response in cells deficient or not for IRF-3, RIG-I, or MDA5 (Fig. S3C and D). However, MOPV_ExoN_ and LASV_ExoN_ induced a type-I IFN response in dHepaRG^CTR^ cells and dHepaRG^MDA5^ cells compared to wild-type viruses but not in cells deprived of IRF3 or RIG-I expression (Fig. S3C and D). These data demonstrated that in the absence of a fully efficient ExoN domain, MOPV and LASV arenaviruses induce the type-I and type-III IFN responses that depend exclusively on the RIG-I/MAVS/IRF3 pathway and that this IFN response may participate in the control of their replication.

### RIG-I binds MOPV and LASV RNA isolated during viral replication, that activate the IFN response

Knowing that RIG-I is the major sensor of MOPV and LASV, we took advantage of HEK293 ST-RIG-I cells overexpressing a one-STrEP-tagged version of RIG-I that previously helped in identifying the RIG-I associated RNA during the replication of several viruses (29–32). We first analyzed the growth kinetics of MOPV_WT_ and MOPV_ExoN_ in HEK293 ST-RIG-I cells and in control HEK293 ST-CH cells overexpressing a one-STrEP-tagged version of mCherry and both cell lines infected at an MOI of 0.1 (Fig. S4). As observed in A549 cells, MOPV_WT_ replicated efficiently, and MOPV_ExoN_ was attenuated by 1,000 times in both cell lines when looking at the quantity of infectious particles released in the supernatants (Fig. S4A). At the RNA level, however, MOPV_ExoN_ produced about 100 times less RNA in the supernatant of infected cells compared to MOPV_WT_ (Fig. S4B), but there was almost no difference in the amount of RNA for both viruses detected in cell lysates (Fig. S4C). Considering the amount of intracellular viral RNA in the infected cells at 24 h during the exponential phase of RNA production, we picked this time point for the collection of RIG-I-associated RNA.

**Fig 4:**
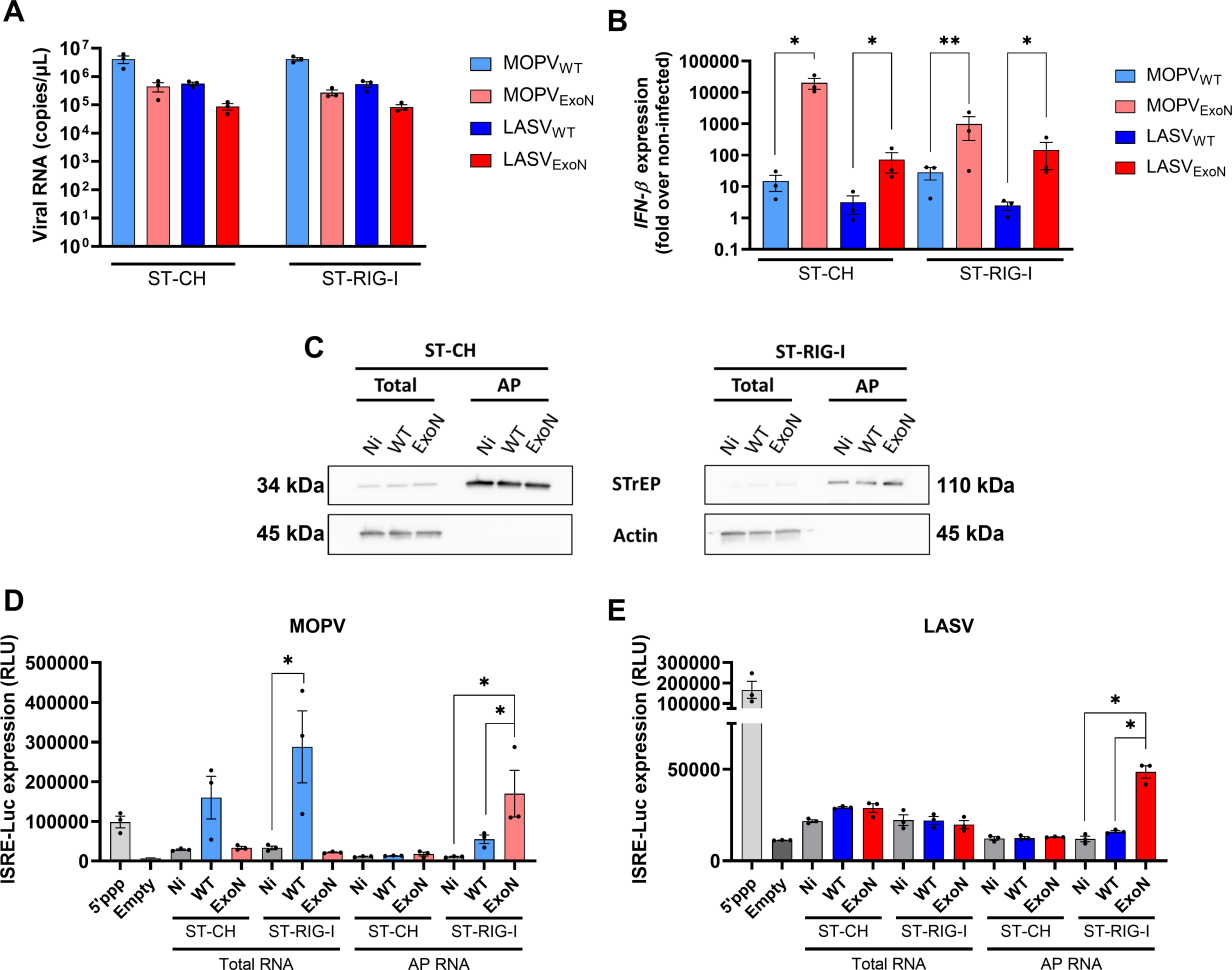
Purification of viral RNA associated with RIG-I during MOPV and LASV infection. HEK293 ST-CH or ST-RIG-I were either mock-infected (non-infected Ni, gray) or infected with MOPV_WT_ (light blue), MOPV_ExoN_ (pink), LASV_WT_ (dark blue), or LASV_ExoN_ (red) at an MOI of 0.1. At 24 h PI, cells were lysed using MOPS buffer and purified using a STREP-Tactin resin. Intracellular viral genome copies were quantified using RT-qPCR targeting the NP of both MOPV and LASV (**A**). The induction of IFN-β was determined via quantification of intracellular RNA using RT-qPCR (**B**). Expression (total) and purification (AP) of the one-STrEP-tagged proteins were verified by western blot using an anti-STrEP-tag antibody. The expression of actin was also verified in the total extracts (**C**). Purified RNA from cells infected or not with MOPV was transfected into HEK293 in-house ISRE reporter cells (**D**), and induction of the interferon response was quantified as relative luciferase units (RLU). As a positive control, dsRNA with a 5′ppp was also transfected into the ISRE reporter cells. RNA from cells infected or not with LASV was transfected into HEK293 STING37 reporter cells (**E**), and induction of the interferon response was quantified as RLU. All figures represent the mean of three biologically independent experiments ± SEM, and statistical significance was determined via Student’s *t*-test (*P < 0.05, **P < 0.01).

HEK293 ST-RIG-I cells and HEK293 ST-CH cells were then infected or not with the WT and ExoN mutant recombinant MOPV and LASV viruses at an MOI of 0.1. At 24 h, cells were lysed, then ST-RIG-I and ST-CH were affinity-purified (AP) through their one-STrEP-tag on a Streptactin column. RNA was then extracted from the total lysates and from the AP samples. Similar amounts of MOPV and LASV RNA were found in the total extracts of HEK293 ST-RIG-I cells and HEK293 ST-CH cells, suggesting that overexpression of RIG-I had no effect on the virus replication (Fig. 4A). Nonetheless, we detected about 10 times less LASV RNA in both cell lines compared to MOPV. We also demonstrated that in both cell lines at 24 h post-infection, the expression of *IFN-β* was significantly induced by MOPV_ExoN_ and LASV_ExoN_ (Fig. 4B). We confirmed that ST-RIG-I and ST-CH were efficiently pulled down by the Streptactin column (Fig. 4C). To test the immunostimulatory activity of the total or ST co-purified RNA, we transfected RNA into a reporter cell line expressing luciferase under the control of an interferon-stimulated response element (ISRE) promoter as described in reference 33. Total RNA from MOPV_WT_-infected cells activated the ISRE promoter, with a higher immunogenicity of total RNA extracted from HEK293 ST-RIG-I cells compared to RNA extracted from ST-CH cells (Fig. 4D). Total RNA from MOPV_ExoN_-infected cells did not activate the IFN response, but RIG-I-AP RNA from both MOPV_WT_ and MOPV_ExoN_-infected cells significantly induced the IFN response, with a higher activation for the MOPV_ExoN_ RNA. CH-AP RNA did not activate the reporter cell line. For LASV (Fig. 4E), only the LASV_ExoN_ RNA co-purified with RIG-I activated the type-I IFN response. These data indicate not only that RIG-I is the principal cytoplasmic receptor responsible for inducing the IFN response during MOPV and LASV replication, but that RNA associated with RIG-I in the context of infection is immunogenic, underlying the critical role a functional ExoN domain plays in preventing host detection of MOPV and LASV PAMPs.

### RIG-I recognizes different regions of the MOPV and LASV RNA

To identify the RNA molecules activating RIG-I during MOPV and LASV replication, we analyzed the total and AP RNA by next-generation sequencing (NGS) of three biological replicates.

We obtained between 14 and 43 million reads for MOPV, with viral reads representing around 0.5% of the total library. This corresponds to genome coverage ranging from 320× for MOPV_ExoN_ to 3,100× for MOPV_WT_. For LASV, we obtained approximately 74 million reads, with viral proportions of about 0.1% for LASV_WT_ and 0.01% for LASV_ExoN_, yielding genome coverage of 400× and 40×, respectively. Sample quality was assessed using mCherry protein expression levels, which led to the exclusion of the third biological replicate of mCherry total samples due to low mCherry expression for both LASV_WT_ and LASV_ExoN_. The raw reads were aligned to the viral genomes, and the coverage was computed position by position along the genome. A sliding window was applied to transform the position-based matrix into a feature-based matrix. Then, each window was normalized using the trimmed mean of M-values (TMM) method from edgeR. A coefficient of variation (CV) filter was applied to remove positions displaying excessive variability between biological replicates. Finally, the normalized AP RNA coverage was divided by the normalized total RNA coverage in the same experiment, and the RIG-I AP/RIG-I total ratio was controlled using the negative control mCherry AP/Cherry total ratio, as described in Chazal et al. (30). This final ratio reflects the specific enrichment of viral RNA with RIG-I.

**Fig 5:**
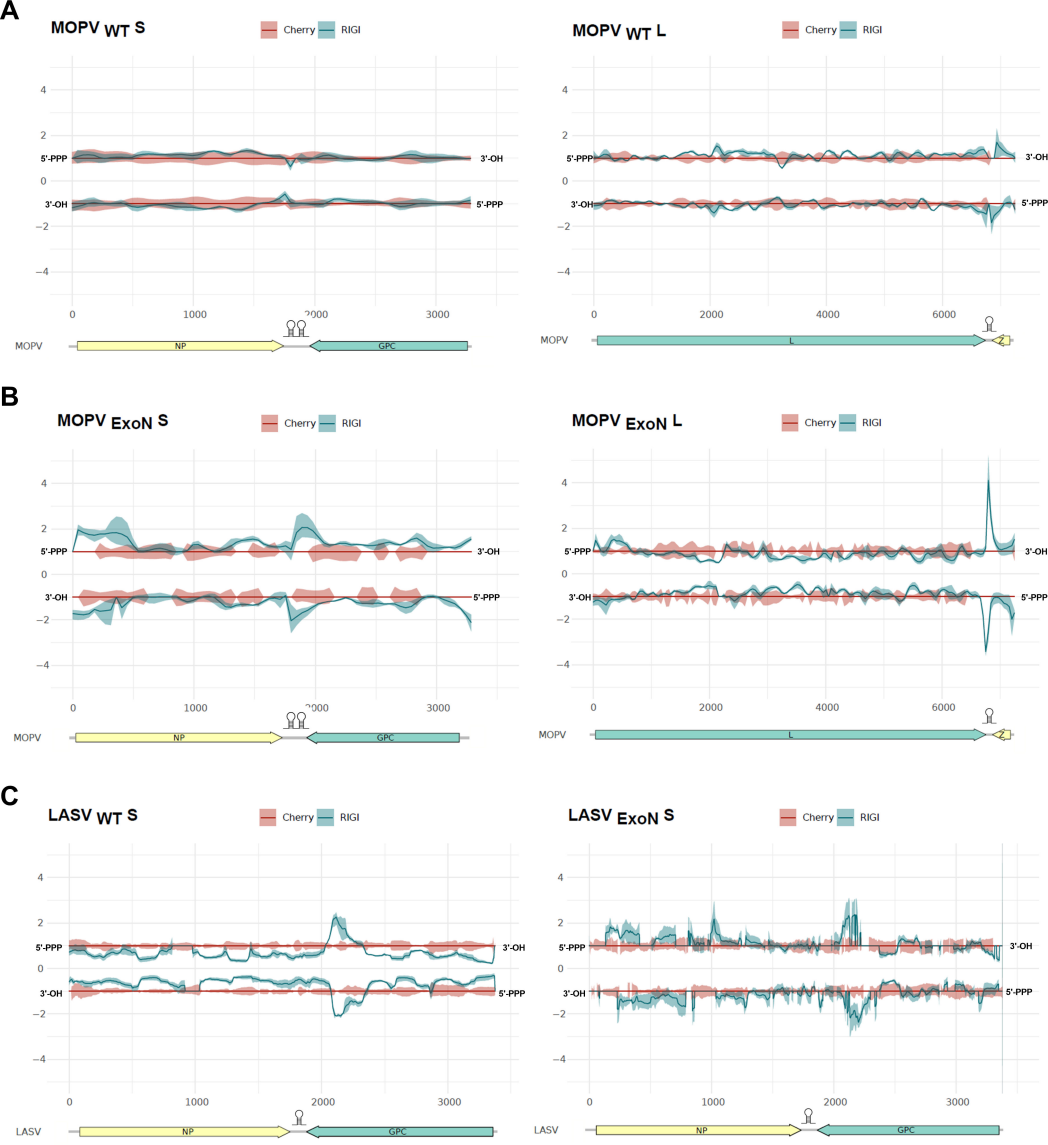
Enrichment of RIG-I-associated viral regions during MOPV and LASV infection. For each strand, the AP mCherry and RIG-I normalized coverages were standardized to the corresponding total samples. The normalized RIG-I coverage was then compared to normalized mCherry coverage (non-specific binding). The x-axis represents the position along the viral segment, and the y-axis shows the fold enrichment in AP between RIG-I and mCherry. Normalized RIG-I binding is plotted as positive values for the positive strand and as negative values for the negative strand. Data are presented for MOPV_WT_ (**A**), MOPV_ExoN_ (**B**), and the S segments of LASV_WT_ and LASV_ExoN_ (**C**). Data represent the mean of three independent biological replicates. The 5′ and 3′ ends of each genomic or antigenomic segment are indicated (5′-PPP or 3′-OH), as well as the localization of the hairpin structures in the intergenic regions.

Based on previous studies, we set up a threshold of 2 for any enrichment that could be biologically significant. In this sense, MOPV_WT_ sequences from AP RNA were not particularly enriched in RIG-I samples compared to the mCherry samples (Fig. 5A). However, several regions of the S and L segments of MOPV_ExoN_ were enriched by about twofold or more in the RIG-I samples (Fig. 5B). These regions included the 5′ termini of the S segment both on the genomic and antigenomic RNA, the 5′ terminus of the genomic L segment, the intergenic sequence of the S segment, and the intergenic sequence of the L segment. The intergenic region of the L segment was particularly enriched in RIG-I samples by more than threefold for the genomic RNA and more than fourfold for the antigenomic RNA. Regarding LASV, we could only analyze the enrichment for the S segment, as viral read coverage was not sufficient to correctly interpret the enrichment on the L segment (Fig. S5). For both LASV_WT_ and LASV_ExoN_ S segments, we only detected an enrichment (>2) of a sequence corresponding to the GPC gene (LasS−GPC and LasS+GPC), both on the genomic and antigenomic strands (Fig. 5C). Therefore, RIG-I still recognizes the LASV GPC RNA in the presence of a functional ExoN domain.

**Fig 6:**
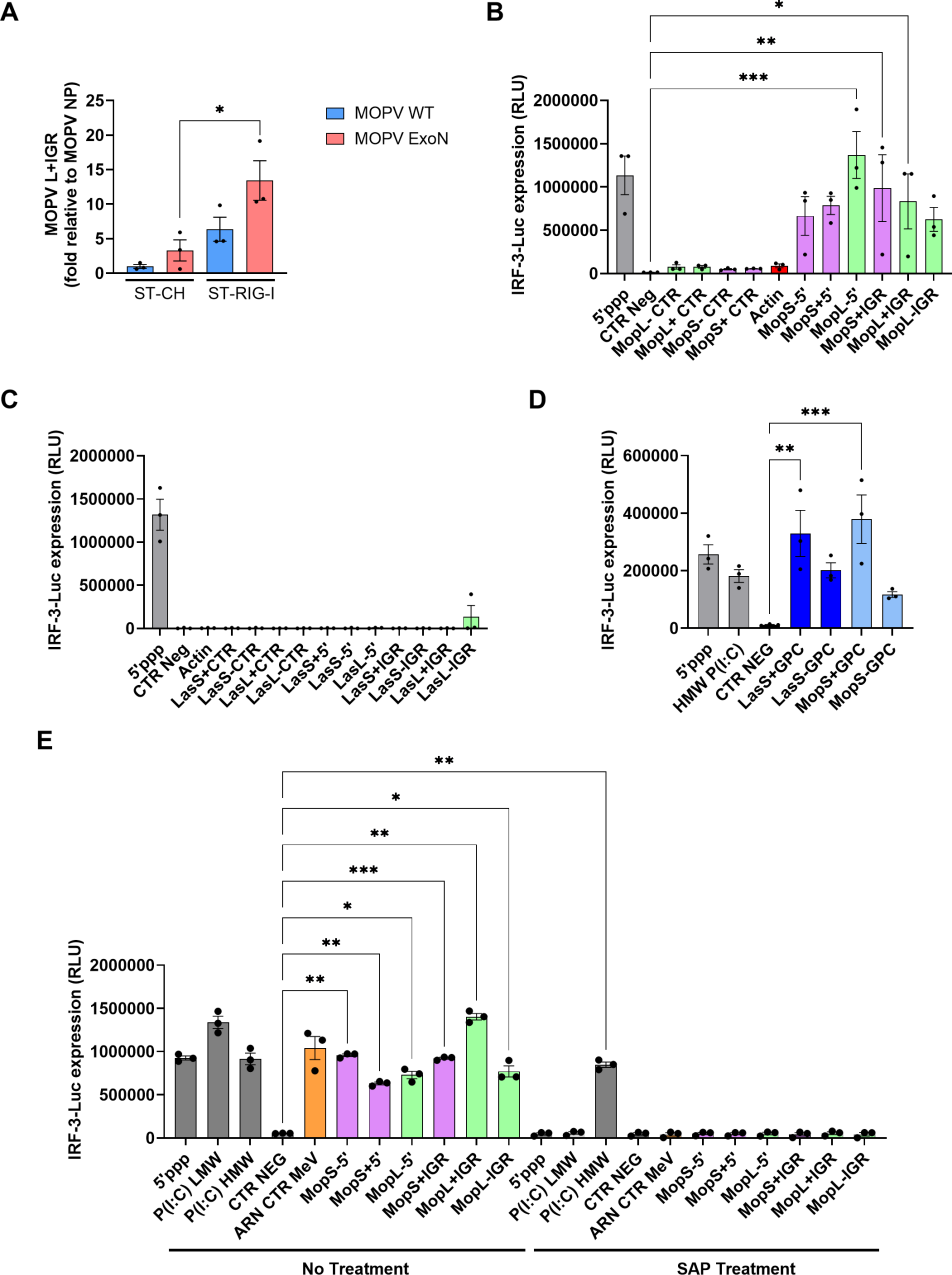
Activation of reporter cell lines by synthetic viral RNA. Based on sequencing data, primer sets, and probes were designed for the highly enriched MOPV Lag sequence observed in Fig. 5A and B. Purified RNAs from the same experiment were analyzed by RT-qPCR to identify an enrichment of these sequences, using MOPV NP as a baseline sequence. Enrichment of the MOPV_WT_ (light blue) and MOPV_ExoN_ (pink) Lag was represented as expression relative to NP (**A**). Synthetic RNAs corresponding to sequences purified during MOPV infection were produced using plasmids coding for viral sequences under T7 promoter control (**B**). These RNAs were then transfected into an A549-DUAL cell reporter, and activation of the interferon response by RNA corresponding to the Sag (purple) or Lag (light green) is represented as RLU. A dsRNA possessing a 5′ppp was used as a positive control (gray), and 200 bp of the human actin sequence was used as an external negative control (red). Corresponding positions on LASV were also produced in plasmids under T7 control, and synthetic RNAs were produced and transfected into an A549-DUAL cell reporter (**C**). Synthetic RNAs corresponding to the enriched LASV GPC and equivalent sequences from the MOPV genome were transfected into an A549-DUAL cell reporter (**D**). Synthetic MOPV RNAs, treated or not with SAP, were transfected into A549-DUAL cells to assess their ability to activate the interferon response (**E**). Activation of the subsequent interferon response is represented as RLU. All figures represent the mean of three biologically independent experiments, and significance was determined via Student’s *t*-test (*P < 0.05, **P < 0.01, ***P < 0.001). The risk of error between each sample is represented as SEM.

We designed RT-qPCR primers and probes to confirm the enrichment of the immunostimulatory RNAs in the AP RNA. Most sequences were too GC-rich to allow proper design of amplifying tools, but we were able to confirm an enrichment of the MOPV L IGR + sequence by about 10-fold in the RIG-I AP RNA compared to the Cherry AP RNA for MOPV_ExoN_, and by about fivefold for MOPV_WT_ (Fig. 6A). To confirm that the enriched sequences activate the IFN response through RIG-I, we cloned the corresponding DNA sequences into plasmids for *in vitro* RNA production, except for the S IGR-sequence, which we could not clone successfully without mutations or deletions. Each synthetic RNA contained the enriched sequence on the 5′ end of the RNA and the following genomic nucleotides, up to 200 bases (Table S1). In this sense, all synthetic RNAs had the same length. We also selected random sequences of 200 bases from the genomic and antigenomic S and L segments (Mop or LasL-CTR, Mop or LasL+CTR, Mop or LasS-CTR, and Mop or LasS+CTR) to produce control RNAs. We then transfected A549 Dual cells and evaluated the induction of the IRF pathway by luciferase measurement. For MOPV, none of the control viral sequences activated the reporter, but all the enriched sequences induced strong expression of the reporter (Fig. 6B). We produced LASV RNA corresponding to the same MOPV-activating sequence. None of these LASV synthetic RNAs activated the reporter (Fig. 6C), suggesting that MOPV and LASV RNAs, corresponding to the same sequences on the viral genomes, possess different affinities toward RIG-I. For LASV, both LasS−GPC and LasS+GPC activated the reporter (Fig. 6D). Unexpectedly, the corresponding MopS−GPC and MopS+GPC on the MOPV genome also activated the IRF pathway (Fig. 6D), suggesting that these RNAs may be equally immunogenic for MOPV and LASV but may not be produced during MOPV replication. Finally, when MOPV synthetic RNAs were SAP-treated before transfection, they no longer induced type-I IFN signaling (Fig. 6E). Remarkably, we noticed some variations in the ability of the different non-treated MOPV RNAs to activate the IRF3 reporter. Overall, our data suggest that ExoN-deficient MOPV and LASV produce different immunogenic RNAs that are recognized by RIG-I during infection. Additionally, our data suggest that MOPV generates more RIG-I-activating RNAs than LASV during viral replication.

## DISCUSSION

In this study, we analyzed the respective roles of RIG-I and MDA5 in the induction of interferon responses by Old-World arenaviruses. A previous study from our team suggested that RIG-I was central to the induction of IFN responses by an ExoN mutant LASV (12), but the role of MDA5 was not deeply investigated at that time. We here confirm that the ExoN-deficient viruses MOPV and LASV activate the IFN response exclusively through RIG-I in A549 and HepaRG cells. We did not directly assess the role of TLR-3 in the induction of the IFN response by MOPV and LASV. TLR-3 is expressed on A549 cells (34) and on HepaRG (27) and is responsible for an independent signaling pathway for the induction of the IFN response upon dsRNA recognition (35). Considering the absence of IFN synthesis in the absence of RIG-I and MAVS, our results strongly suggest that TLR-3 is not relevant in our *in vitro* infection model. While the role of MDA5 has not been deeply evaluated *in vitro*, several studies suggested that MDA5 could play a role in the induction of IFN responses during arenavirus infections *in vivo*, in addition to RIG-I. Indeed, MAVS KO and MDA5 KO mice don’t produce type-I IFN following infection with LCMV, while WT mice do (36). Similarly, PICV virus replicates more efficiently in MDA5 KO and MDA5/RIG-I DKO mice than in WT mice without inducing type-I IFN responses (14). However, LCMV, PICV, MOPV, and LASV may produce different RNAs during their replication, with different abilities to activate RIG-I or MDA5. Indeed, dsRNA can be detected in cells infected with LCMV, JUNV, and MACV using mAb 9D5 (37–39). Of note, Mateer et al. failed to visualize dsRNA in LASV-infected cells using the mAb 9D5 antibodies (39), and several attempts from our lab to detect such dsRNA in cells infected with WT or ExoN MOPV and LASV were also unsuccessful, with mAb 9D5 or J2 antibodies. However, mAb 9D5 does not react efficiently with dsRNA shorter than 100 bp (37), and J2 only recognizes perfect RNA duplexes longer than 40 bp (40). The RNA molecules produced during MOPV and LASV infections and activating RIG-I that we have identified in our study are short (between 10 and 191 nucleotides long) and may not form perfect RNA duplexes, probably explaining the lack of reactivity with mAb 9D5 and J2.

Interestingly, MOPV_ExoN_ and LASV_ExoN_ mutant viruses were more attenuated in A549 cells than in 293T cells, HeLa cells, or HepaRG cells. This may be due to the more robust IFN response observed in A549 cells compared to other cell lines. Indeed, type-I IFN expression was increased by 4 log in the A549 cells following ExoN infection, while it was only induced by 2 log in the HepaRG cells at the same time point using the same MOI. A549 cells are known to express high endogenous levels of RIG-I (41), and this may explain the difference in type-I IFN response and its effect on virus replication depending on the cellular model used. In primary human macrophages, both MOPV and LASV_ExoN_ also strongly activate the type-I IFN response and are consequently strongly attenuated (8, 25). Altogether, these data suggest a dose-dependent effect of IFN on the replication of arenaviruses.

While the ExoN domain of LASV and MOPV is crucial for the evasion of the IFN response, it may also be essential for transcription and replication. Indeed, we previously demonstrated that mutating the ExoN domain of LASV affected the ability of NP to participate in the transcription/replication of a LASV minigenome (8). This may explain why MOPV_ExoN_ and LASV_ExoN_ never replicate to the levels of wild-type counterparts in the absence of an IFN response in MAVS or RIG-I KO cells, or in Vero cells (8, 25).

Remarkably, both WT MOPV and LASV viruses induce little to no type-I IFN response and are not attenuated in A549 cells, while WT MOPV significantly activates the type-I IFN response in primary macrophages (25, 42). This discrepancy may be explained by the quality of the antiviral response in macrophages compared to epithelial cells. Indeed, macrophages express higher levels and a larger diversity of PRRs detecting non-self RNA and were proposed to express more ISGs following IFN stimulation than other cell types, particularly upregulating ISG-encoding pathogen recognition receptors (43). It is therefore possible that RIG-I is more rapidly and specifically expressed in macrophages than in epithelial cells, thus leading to faster control of RIG-I-activating viruses. Nevertheless, we observed a trend toward higher activation of the IFN response by MOPV_WT_ than by LASV_WT_ in A549 cells, even if the difference was not significant compared to non-infected cells. We also observed differences in viral kinetics in A549 cells between LASV and MOPV. If LASV does indeed replicate quicker than MOPV, it is likely that the strict control of type-I IFN observed by LASV is due to the rapidity with which NP is produced and therefore available to control cellular responses. Our data suggest that MOPV produces more PAMPs than LASV. Indeed, RIG-I-associated RNA derived from both WT and ExoN MOPV viruses activated the IFN response, while only LASV_ExoN_ RNA was immunogenic. In addition, several RNA sequences from MOPV were observed to activate RIG-I, while only one LASV sequence could activate RIG-I. LASV may therefore be intrinsically less immunogenic by producing less RIG-I-activating RNA and rapidly expressing large amounts of NP.

The RIG-I-activating RNAs produced during MOPV replication corresponded to the extremities and intergenic regions of the genomic and antigenomic viral RNAs. Extremities of the genomic segments are likely to form panhandle dsRNA structures due to the high complementarity of their 5′ and 3′ extremities, and such structures seem essential for viral RNA synthesis (44). While these panhandle structures could serve as substrates for RIG-I, Garcin, Kolakofsky, and colleagues demonstrated that the non-templated extra G added by a “prime and realign mechanism” at the 5′ extremity of the genomic segments prevented their recognition by RIG-I (23, 45, 46). It remains to be determined whether the enriched genome extremities that we observed in the RIG-I precipitates correspond to these panhandles. Indeed, synthetic RNAs corresponding to one strand still activated the IFN response, suggesting that the 5′ extremities of the MOPV genome rather form secondary structures recognized by RIG-I. This activation was completely dependent on the presence of a 5′ triphosphate. The IGR of arenaviruses and other *Bunyaviricetes* are known to form stable dsRNA hairpin structures between the two genes on each genomic and antigenomic segment (47). The IGR sequence acts as a transcription stop signal for the synthesis of viral mRNA, where the viral polymerase pauses to terminate mRNA synthesis (48). In this sense, all arenavirus mRNA transcripts contain a 3′ structure corresponding to a part of the intergenic region that could act as RIG-I ligands, similarly to the 3′ untranslated regions of Hepatitis C virus and Chikungunya virus (29, 49). It is also possible that while stalling on the IGR transcription stop signal, the polymerase may produce small IGR-like RNAs containing 5′ triphosphate that could activate RIG-I. We confirmed that the MOPV L IGR was enriched in both the mCherry and RIG-I precipitates from MOPV_ExoN_ infected cells.

More surprising is the absence of RIG-I binding and IFN activation by the corresponding LASV genome extremities and IGR sequences. This suggests that the LASV sequences do not have the same conformation and therefore constitute poor RIG-I ligands, despite strong sequence similarities. We attempted to visualize the secondary structures formed by the MOPV and LASV RNA using RNAfold (http://rna.tbi.univie.ac.at/) (Fig. S6). The MOPV 5′ L− and 5′ S+ RNA of MOPV form longer dsRNA stretches with no bulge, while the LASV 5′ L− and 5′ S+ RNA present bulges. The presence of a bulge may dampen the immunogenicity of the LASV RNA, as previously proposed for arenavirus RNAs (23). The MOPV S IGR is also twice the size of LASV S IGR and has been proposed to form two hairpin structures (1), which could explain its higher immunogenicity. However, the MOPV and LASV L IGR are similar in size, and we cannot explain why only the synthetic MOPV L IGR activates the IFN response through RIG-I. We previously collected data suggesting that the IGR of the MOPV S segment was more immunogenic than the IGR of the LASV S segment. Indeed, we generated chimeric LASV and MOPV viruses by swapping the S IGR (26). The chimeric LASV carrying the S IGR of MOPV and this virus was more immunogenic than LASV_WT_ in co-cultures of mDCs with T cells. Conversely, the MOPV virus carrying the S IGR of LASV was less immunogenic than MOPV_WT_ in this model. It will be of interest to generate chimeric LASV and MOPV viruses with swapped L IGR to confirm our new observations regarding the MOPV L IGR. We also identified a sequence in the GPC gene that was enriched in the RIG-I precipitates of LASV_WT_ and LASV_ExoN_-infected cells without activating the IFN response, at least for LASV_WT_. This suggests that the ExoN domain of LASV NP cannot efficiently prevent the recognition of the GPC RNA by RIG-I, but that NP is still able to block the IFN response downstream of RIG-I. Interestingly, the nucleoproteins of several arenaviruses, including LASV, interact with the kinase domain of IKKε to prevent the phosphorylation of IRF3, and the IKKε-binding domain on NP overlaps with the ExoN domain (50). Therefore, mutating the ExoN domain may prevent both the ExoN activity on RNA and the binding to IKKε. In this sense, if LASV_WT_ cannot block the GPC RNA recognition by RIG-I, it can still block IKKε downstream to prevent the activation of the IFN response. However, LASV_ExoN_ can no longer block both the GPC RNA recognition by RIG-I and the activity of IKKε. To our surprise, the corresponding synthetic GPC RNA activated the IFN response for both LASV and MOPV in transfected cells. It is rather difficult to reconcile these observations at this point, and our future research will concentrate on understanding these differences.

The MOPV and LASV NP may also present differences in their ability to digest dsRNA. While previous studies suggest that they both inhibit the IFN response following dsRNA stimulation (25), their intrinsic exonuclease activities have never been compared *in vitro* using recombinant proteins. However, we have demonstrated in a separate study that an MOPV encoding the LASV NP could control the IFN response more efficiently than MOPV_WT_ in infected dendritic cells, and that a LASV recombinant virus harboring the MOPV IGR in the S segment was more immunogenic than its WT counterpart in the same model (26). Therefore, both the exonuclease activity of the NP and the nature of the RNA produced during replication may explain the differences in immunogenicity of MOPV and LASV *in vivo*. Future studies should aim at comparing the ExoN activities of MOPV and LASV NP *in vitro* using recombinant proteins or in infected cells through the generation of chimeric LASV and MOPV viruses with swapped ExoN domains. The 3′→5′ ExoN activity of NP is likely to reduce RIG-I sensing through degradation of viral RNA. *In vitro* studies by Qi et al. (5) or by Hastie et al. (6) proposed that the ExoN activity digests blunt-ended or overhang dsRNA containing or not a 5′ triphosphate. The NP ExoN domain does not seem to require a specific sequence, as it can also block the activation of the IFN response by transfected poly(I:C) or by Sendaï virus infection. We have also demonstrated that the LASV NP could block the IFN induction by a measles vaccine strain in human primary cells (51). It would be interesting to determine if the transfected dsRNA or the Sendaï and measles RNA are actually digested by NP in transfected or infected cells. Examining the ability of the ExoN domain to block the MDA5-dependent activation of the IFN response by natural or artificial RNA would also be interesting.

Notably, the MOPV-derived synthetic RNAs are highly immunogenic *in vitro*. The potential use of these short, immunogenic RNAs to act as immunostimulants, for example, as an adjuvant for vaccines where the RNA induces more potent innate immune activation irrespective of the pathogen of interest, is promising. RIG-I-inducing adjuvants have already been described or proposed for augmenting immune responses to pathogens, such as against H5N1 (52) and antigens produced by recombinant measles virus platforms (53). The addition of MOPV synthetic RNA to existing vaccine formulations could see profound increases in vaccine efficacy, and could therefore represent a target for the future of immunotherapy. Such RNA may also naturally participate in the immunogenicity of MOPEVAC-based vaccines that have been successfully tested in non-human primates against Lassa, Machupo, and Guanarito (51, 54).

In summary, we have identified RIG-I as the primary cytoplasmic receptor inducing the type-I and type-III IFN response *in vitro* in response to LASV and MOPV PAMPs generated during infections by ExoN-deficient viruses. We have identified different regions of the viral genome that specifically activate RIG-I and identified notable differences between MOPV and LASV that could explain, at least in part, their difference in pathogenicity. These data reinforce the central role of the NP in the pathogenicity of arenaviruses and support the development of antivirals targeting the exonuclease domain of NP for the control of arenavirus infection.

## MATERIALS AND METHODS

### Cells and viruses

Vero E6 (African Green Monkey kidney epithelial cells) were used for amplification and titration of LASV and MOPV. A549 (human lung adenocarcinoma epithelial cells) cells were purchased from ATCC. A549^CTR^ and A549^MAVS^ cells were generated by transfection of CRISPR-Cas9-expressing knockout plasmid (control plasmid CRISPR/Cas9 or MAVS: sc-400769-ko-2, Santa Cruz). The KO plasmids are a mixture of three plasmids, each carrying a different guide RNA specific for the target gene, as well as the Cas- and GFP-coding regions. Single-clone selection of GFP-positive cells was performed, followed by validation using western blotting (anti-MAVS, Cell Signaling #3993) and Sanger sequencing of the genomic loci targeted by the guide RNAs. A549^RIG-I^ and A549^MDA5^, KO for RIG-I and MDA5, were kindly provided by Balaji Manicassamy (University of Iowa) and generated as described elsewhere (55). HEK293 in-house ISRE reporter (STING37), HEK293 ST-CH, and HEK293 ST-RIG-I were generated as described elsewhere (29, 33). Vero E6, HeLa, 293T, A549, and HEK293 cell lines were maintained in GlutaMAX Dulbecco’s modified Eagle’s medium (DMEM; Life Technologies) supplemented by Fetal Calf Serum (5% for VeroE6 and HeLa cells, 10% for 293T, A549, and HEK293 cells) and 0.5% Penicillin-Streptomycin at 37°C, 5% CO_2_. A549-Dual cell medium was also supplemented with 0.1% Blasticidin (Invivogen; ant-bl-05) and Zeocin (Invivogen; ant-zn-05). HepaRG cell lines were maintained as previously described (27, 28, 56).

MOPV and LASV recombinant viruses were generated by reverse genetics as described previously (25). MOPV_WT_ is identical to the “wild-type” MOPV strain AN21366, and MOPV_ExoN_ differs from MOPV_WT_ by two NP substitutions, D390A and G393A, which abrogate NP ExoN activity (25). The LASV_WT_ used for this study is identical to the strain AV of LASV, and LASV_ExoN_ differs from LASV_WT_ by two NP substitutions: D389A and G392A (8). Viral stocks were prepared and titrated in Vero E6 cells as previously described (8, 26). For viral titration by focus-forming assay, Vero E6 cells were infected with 10-fold serial dilutions of viral stock in 2% DMEM, then incubated for 7 days at 37°C with carboxymethyl cellulose mixed 1:1 with 5% DMEM. After 7 days, cells were fixed, permeabilized, and stained using rabbit polyclonal anti-Z antibodies and PA-conjugated goat polyclonal anti-rabbit IgG (Sigma-Aldrich). 1-Step NBT/BCIP with suppressor substrate (Thermo Fisher Scientific, Waltham, MA) was used to stain viral foci. Results for titration are expressed as FFU/mL.

### RNA isolation

For qPCR experiments, total cellular RNA was isolated from mock-infected or infected cells by using the RNeasy Minikit (Qiagen) according to the manufacturer’s instructions, and a supplementary DNase step was added using the Turbo DNA-free kit (Ambion; Thermo Fisher Scientific).

For RNA sequencing studies, RNA was obtained from cell lysate using TRIzol LS (Thermo Fisher Scientific) according to the manufacturer’s instructions, and degradation of RNA was impeded using RNasin (Promega).

### Reverse transcription and quantitative PCR

Synthesis of cDNA was performed by using SuperScript III (Applied Biosystems, Thermo Fisher Scientific). Amplification of cDNA was performed by using the gene expression master mix kit (Applied Biosystems, Thermo Fisher Scientific). For IFN-β, the primer/probe mix was developed in-house as described previously (57). IFN-λ (ThermoFisher Scientific; Hs00601677_g1), Mx1 (ThermoFisher Scientific; Hs00895608), and GAPDH (Applied Biosystems; 4325792) were all industrial primer/probe mixes. Runs of qPCR assays were performed with a LightCycler 480 instrument (Roche Diagnostics). The expression levels of all cellular genes were normalized to that of the glyceraldehyde-3-phosphate dehydrogenase (GAPDH) gene and expressed as expression relative to GAPDH. For viral genome quantification, viral RNAs were extracted from culture supernatants or cell lysates using the QIAamp Viral RNA Mini Kit (Qiagen) or the RNeasy Minikit (Qiagen), respectively. Viral genomes were quantified using the SensiFAST Probe No-ROX One-Step Kit (Meridian BioScience, London, UK) and primers and probes targeting MOPV NP (forward 5′-GTCAAGCGTTCTTTGGGAATG-3′; reverse 5′-TCCAGAAAGACATAGTTTGTAGAGG-3′; probe 5′-FAM-TTCCTTTCCCCTGGCGTGTCA-BHQ1-3′) or LASV NP (forward 5′-CTCTCACCCGGAGTATCT-3’; reverse 5′-CCTCAATCAATGGATGGC-3′; probe 5′-FAM-GAACATCCCAAGAGCCC-BHQ1-3′). Viral genome copies were determined using a standard curve generated from dilutions of synthetic viral RNA of known concentrations corresponding to coding fragments of the MOPV and LASV NP gene (including the sequence amplified by PCR) and the equation y = ax + b, where y = Ct, a = slope, x = log concentration, and b = Y-intercept. All runs were performed in duplicate using a LightCycler 480 (Roche).

### Affinity chromatography of RLR RNP complexes and subsequent RNA and protein purification

Cells overexpressing ST-proteins were seeded at 18 × 10^6^ per T175cm flask (ThermoFisher). Twenty-four hours after seeding, these cells were either mock-infected or not with MOPV_WT_, MOPV_ExoN_, LASV_WT_, or LASV_ExoN_ at an MOI of 0.1. After 24 h PI, cells were detached from flasks using ice-cold PBS and pelleted twice to remove trace amounts of culture medium. Cells were then lysed in MOPS lysis buffer (20 mM MOPS-KOH, 120 mM KCl, 0.5% Igepal, 2 mM MgCl₂, 2 mM BME supplemented with protease and RNase inhibitors) for 20 min at 4°C, before high-speed centrifugation to pellet cellular debris. A 100 µL of cell lysate was kept for total RNA extraction, and the remaining lysate was incubated with a STREP-Tactin resin (IBA Lifesciences) for at least 2 h at 4°C. The resin was then centrifuged for 5 min before washing three times with MOPS washing buffer (see MOPS lysis buffer, without addition of Igepal). Proteins were disassociated from the resin using Elution Buffer (IBA Lifesciences), in three 15-min intervals at 4°C. Total and purified RNA extractions were performed using TRIzol. For RNA extraction, the cell lysate was mixed with Trizol LS (ThermoFisher Scientific) at a ratio of 3:1, and 20% of the subsequent volume in chloroform was then added to each sample. The samples were vortexed for 30 s and left for 5 min at RT before centrifugation at 13,200 rpm for 15 min at 4°C. The upper aqueous phase of the resulting separation was collected, and 0.5% of the volume in glycogen (5 µg/µL stock) was added to each sample and vortexed well. One volume of the sample in RT isopropanol was then added to each sample and vortexed well. The samples were then kept overnight at −20°C. The samples were centrifuged at 20,000 × *g* for 30 min at 4°C, and the supernatant was discarded. Pellets were washed in 70% ice-cold ethanol, vortexed for 10 s, and once again centrifuged at 20,000 × *g* for 15 min at 4°C. The supernatant was discarded, and pellets were left to dry in an RNase-free environment for no longer than 3 min. After this time, if any remaining ethanol was present, it was removed with a pipette. The purified RNA was then resuspended in sterile, DNase and RNase-free water, and the samples were left on ice for 30 min and occasionally vortexed. Finally, samples were aliquoted in small working volumes for quantification and analysis to avoid multiple freeze-thaw cycles.

A western blot was performed to confirm the expression of the ST proteins in the whole cell extracts and their purification in the affinity-purified samples. The concentration and quality of the extracted RNA were verified using a DeNovix spectrophotometer (DeNovix Inc.) and a TapeStation (Agilent).

### Western blot

Cell lysates were separated by SDS-PAGE on 4%–20% Criterion gels (Biorad). Proteins were transferred to a polyvinylidene difluoride membrane using a Mini Trans-Blot electrophoretic transfer cell apparatus (BioRad). Membrane staining was performed using specific antibodies to RIG-I (Cell Signaling; 4200), MDA5 (Abcam; ab69983), MAVS (Cell Signaling; 3933), or Streptavidin conjugated to HRP (Biorad; STAR5B) and HRP-conjugated secondary antibodies (Jackson Laboratories; 111-035-144). Actin was detected using HRP-conjugated antibodies (Sigma; A3854). Staining was revealed using Clarity ECL substrate (BioRad) and imaged using an ImageQuant LAS4000 imager (GE).

### Next-generation sequencing

For LASV samples, the quality of samples was checked by TapeStation (Agilent), and RNA was quantified by Nanodrop. First, 100 ng of total RNA was depleted of rRNA (MGIEasy rRNA depletion kit), then library preparation was realized with the MGIEasy RNA Directional Library Prep Set (MGI). The quality of libraries was checked by TapeStation (Agilent) and quantified by Qubit 1X dsDNA HS Assay Kit. Samples were pooled, and after circularization in ssDNA, DNB (DNA NanoBalls) were made, following the manufacturer’s protocol. Then sequencing was performed on the MGI DNBSEQ-G400, running Paired-End 300 bp on a Flow Cell Large PE150 (MGI). Data were demultiplexed with BasecallLite v1.5.0.323.

For MOPV samples, protocols for NGS library preparation and NGS of total and RLR-bound RNA have been described previously (29, 30). Depletion of ribosomal RNA was performed for INPUT and OUTPUT samples using the riboZero reagents included in the TruSeq stranded total RNA library prep kit (20020596, Illumina). NGS libraries were generated following the manufacturer’s protocol. The indexed samples were multiplexed to obtain approximately 25 million reads per sample. Sequencing was performed on the Illumina NextSeq500 platform to generate single-end 65 bp reads bearing strand specificity.

### Read preprocessing

Sequencing reads underwent thorough preprocessing to ensure the removal of adapter sequences and low-quality reads. This process was executed using cutadapt version 2.10. Following this, reads with a minimum length of 25 nucleotides were retained for downstream analysis. Alignment to the human reference genome (GRCh38 from Ensembl version 104) was performed using STAR version 2.7.9a with default parameters, complemented by samtools version 1.18. Reads are also aligned to the MOPV reference genome (AN21366) and the LASV reference genome (FR832710.1 and FR832711.1) using bowtie2 version 2.3.5.1 in its very sensitive mode.

### RLR binding analysis

MOPV and LASV coverage analysis was conducted using BEDTools version 2.29.2 with the “genomecov -d” parameters. Data analyses were conducted in R version 4.2.2, leveraging bioconductor packages, such as ggplot2 version 3.4.2 and tidyverse 2.0.0. Position-wise coverage matrices were transformed using a sliding window approach implemented with the zoo R package (version 1.8-14). A window size of 100 bp with 50 bp overlap was applied to the viral genome. For each window, read counts were summed across constituent positions. Normalization was performed using the TMM method from the edgeR package (version 4.6.3). Raw counts were then scaled by the calculated scaling factors to obtain normalized coverage values. For each genomic position, CV was calculated within the three biological replicates as CV = σ/μ × 100, where σ is the standard deviation, and μ is the mean coverage. We empirically tested CV thresholds from 20% to 60%. The 40% threshold was selected as the optimal middle ground. It effectively filtered out the most inconsistent genomic regions while retaining signals that are consistent across replicates, ensuring that the reported results are robust and reproducible. RIG-I binding was calculated following the methods outlined in Chazal et al. (30). The coverage of bead samples was normalized by the mean coverage of the corresponding total sample. Subsequently, the normalized bead samples for each RIG-I were further normalized by the mean coverage of the triplicates for mCherry samples at each genomic position to determine RIG-I binding. Visualization of RLR binding for each condition was accomplished using ggplot2.

### Luciferase assays in reporter cell lines

The HEK293 in-house ISRE reporter cell line was seeded in 24-well plates (Corning, Fisher Scientific) at a density of 6 × 10^6^ cells per plate. Cells were transfected with 100 ng of total or purified RNA, mixed with Lipofectamine 2000 (Invitrogen; ThermoFisher). After 24 h, cells were lysed with a Passive Lysis Buffer (Promega) and lysate mixed with the Bright-Glo Luciferase Assay system (Promega) for analysis using an Infinite Plate Reader (Tecan).

For synthetic RNA studies, A549-DUAL cell lines were seeded in 24-well plates (Corning, Fisher Scientific) at a density of 4.8 × 10^6^ cells per plate. Cells were transfected with 10 ng of synthetic RNA mixed with JetPrime transfection reagent (Polyplus). After 18 h, the cell supernatant was collected and mixed with QuantiLuc 4 Lucia/Gaussia Luciferase Assay system (InvivoGen) for analysis using an Infinite Plate Reader (Tecan).

### Production of synthetic RNA

5′ppp control for RNA studies was bought from Invivogen (tlrl-3prna), as well as poly(I:C) LMW (tlrl-picw) and HMW (tlrl-pic). We chose 200 bp sequences of relevant MOPV or LASV genomes using sequencing data, which were inserted into the commercially available P2RZ (Addgene). Plasmids were linearized using the Xho-1 restriction enzyme before RNA was synthesized using the T7 RiboMAX Express Large Scale RNA Production System. The resulting RNA was subjected to a Wizard DNA Cleanup protocol (Promega) to remove any residual plasmid DNA and then additionally passed through an RNA cleanup protocol using RNeasy RNA purification columns (Qiagen). This final RNA product was quantified and purity assured using Nanodrop and TapeStation (Agilent).

### Statistical analysis

Data were analyzed using the analysis tool in PRISM (Version 10, GraphPad Software). Unless otherwise stated, results are shown as means ± SEM from three independent experiments. Significance was calculated using Student’s *t*-test for pairwise comparisons or one-way ANOVA with a Tukey’s multiple comparison test when comparing three or more sets of values. Statistically significant differences are represented as follows: *P < 0.05; **P < 0.01; ***P < 0.001.

## Data Availability

The authors confirm that the data supporting the findings of this study are available within the article. The sequencing data used in this publication are available in the European Nucleotide Archive (https://www.ebi.ac.uk/ena) under accession number PRJEB101864.

## SUPPLEMENTAL MATERIAL

Supplemental figures jvi.02110-25-s0001.pdf Fig. S1 to S6.

Table S1 jvi.02110-25-s0002.xlsx Cloned cDNA sequences for the *in vitro* production of control and arenavirus-derived RNA.

ASM does not own the copyrights to Supplemental Material that may be linked to, or accessed through, an article. The authors have granted ASM a non-exclusive, world-wide license to publish the Supplemental Material files. Please contact the corresponding author directly for reuse.
