# Supplementary material for: Lassa and Mopeia viruses produce different RIG-I-activating RNA in the absence of a functional viral exoribonuclease domain
Source: J Virol. 2026 May 11;100(6):e02110-25. doi: 10.1128/jvi.02110-25 (PMC13288612; doi:10.1128/jvi.02110-25)
Supplement: Supplemental figures — Fig. S1 to S6. [file jvi.02110-25-s0001.pdf]

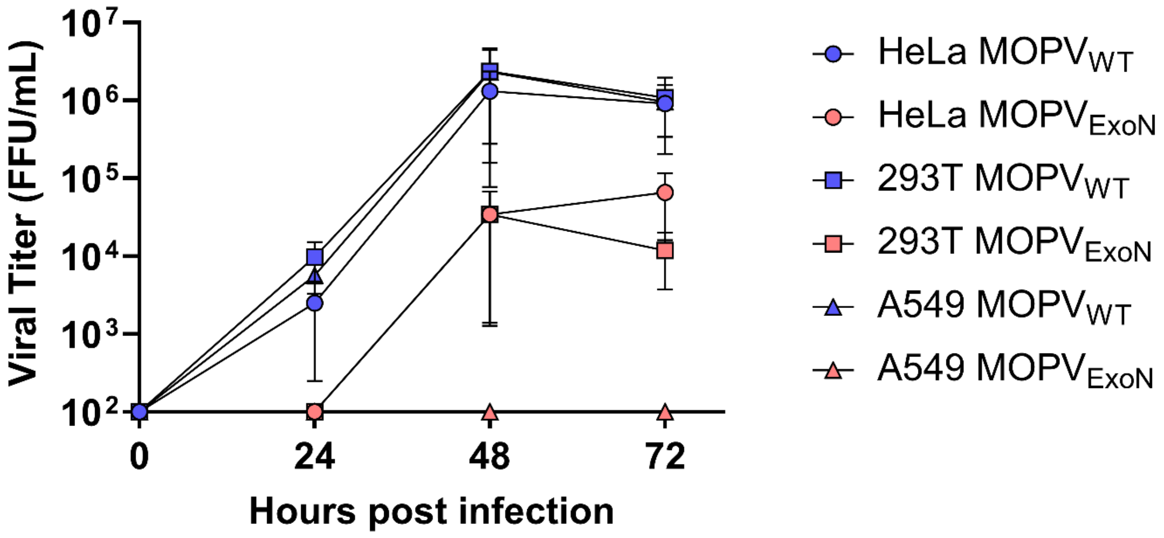

Figure S1. Replication of MOPV$_{WT}$ and MOPV$_{ExoN}$ in IFN-competent cell lines. HeLa, 293T and A549 cells were infected by recombinant MOPV$_{WT}$ (light blue) and MOPV$_{ExoN}$ (pink) at MOI 0.01. Cell supernatant was collected at 24h intervals and analyzed by Focal Forming Assay (FFA). Mean values represent three biologically independent experiments, with error represented as standard error of the mean (SEM).

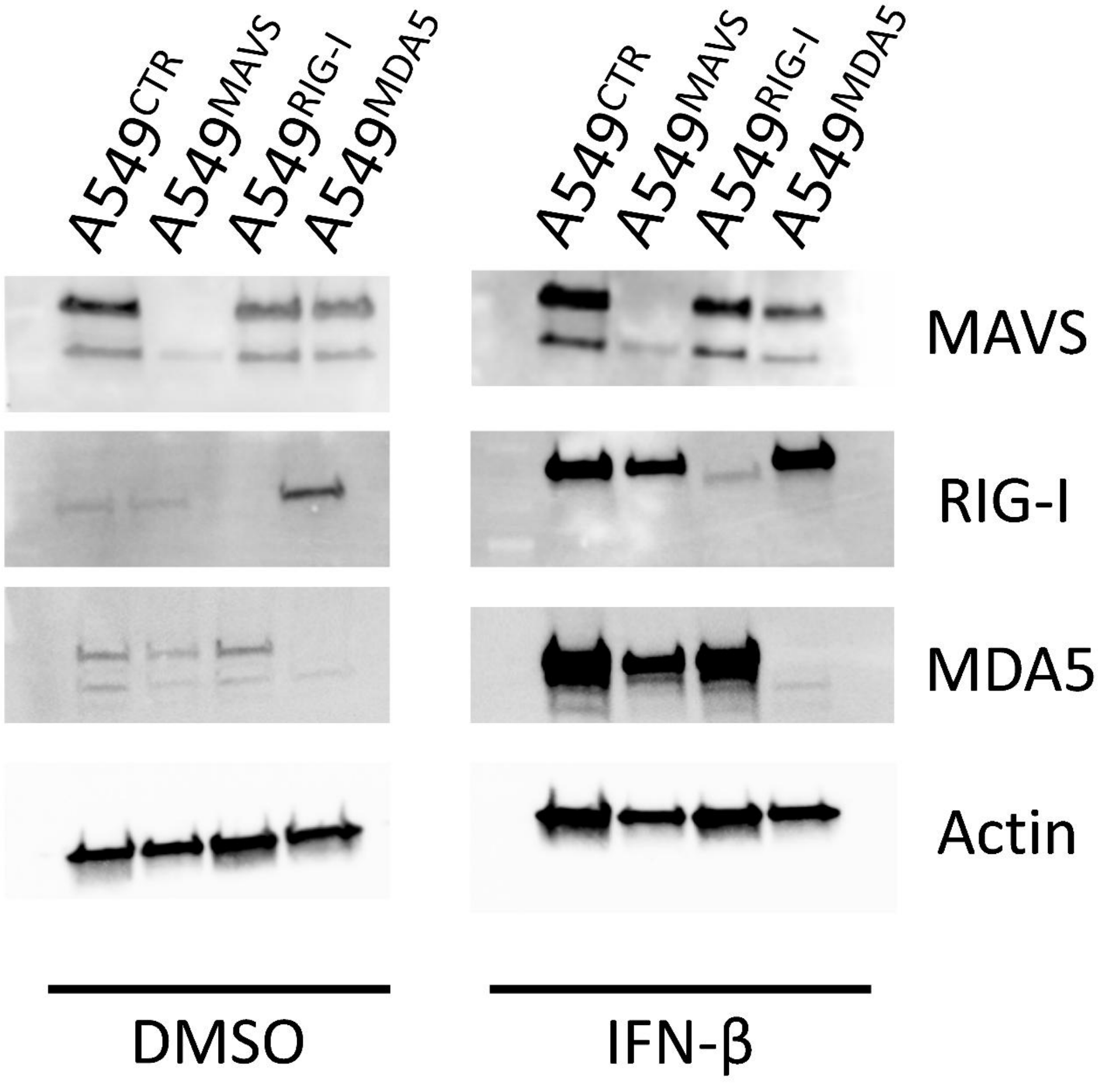

Figure S2. Expression of MAVS, RIG-I and MDA5 in the A549 cell lines. A549^{CTR}, A549^{MAVS}, A549$^{RIG\text{-}I}$ and A549^{MDA5} cells were treated with DMSO or IFN-β (100U/mL) for 48hrs. After 48hrs, cells were lysed in Laemmeli Buffer and protein expression was analyzed by Western Blot.

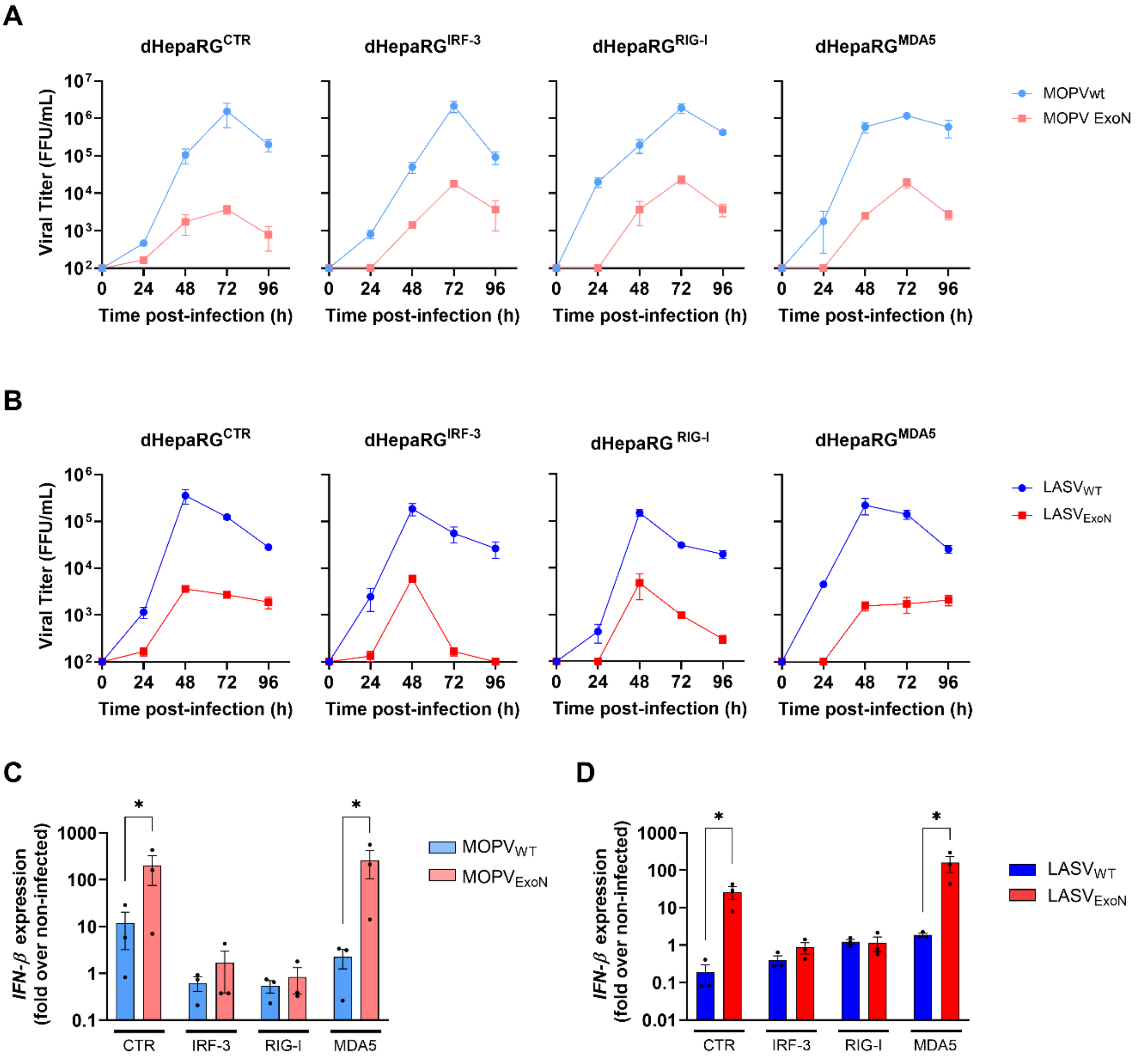

Figure S3. Replication of MOPV and LASV and induction of IFN in dHepaRG cells. Control dHepaRG cells or dHepaRG cells deficient for IRF3, RIG-I or MDA5 were infected with either (A) $MOPV_{WT}$ (light blue), $MOPV_{ExoN}$ (pink) or (B) $LASV_{WT}$ (dark blue) or $LASV_{ExoN}$ (red) and viral titers were determined at different time points post infection. The induction of IFN-β mRNA expression during MOPV infection (C) and LASV infection (D) was determined at 48h PI in the dHepaRG cell lines by qPCR on reverse transcribed cellular RNA. All figures represent the mean ± SEM of three biologically independent experiments and statistical significance was determined via Student T-Test (* $p<0.05$, ** $p<0.01$, *** $p<0.001$).

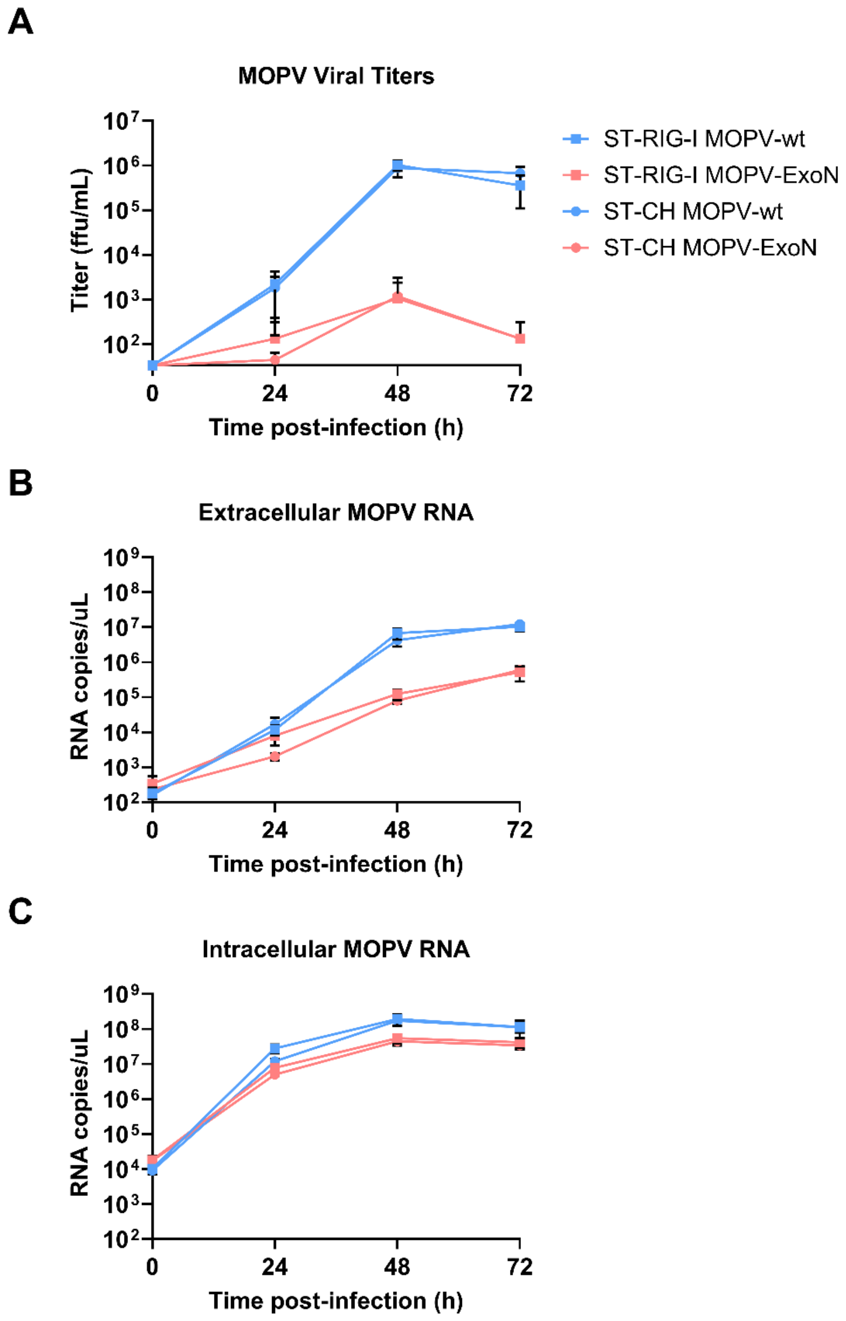

Figure S4. Replication of MOPV in one-STrEP-taggedHEK293 cell lines. HEK293 ST-CH or HEK293 ST-RIG-I were infected with either $MOPV_{WT}$ (light blue) or $MOPV_{ExoN}$ (pink). Cell supernatant was collected and viral titers were analyzed by FFA (A). RNA was also extracted from supernatant (B) and infected cell lines (C) and MOPV RNA was quantified via RT-qPCR. All figures represent the mean of three biologically independent experiments ± SEM.

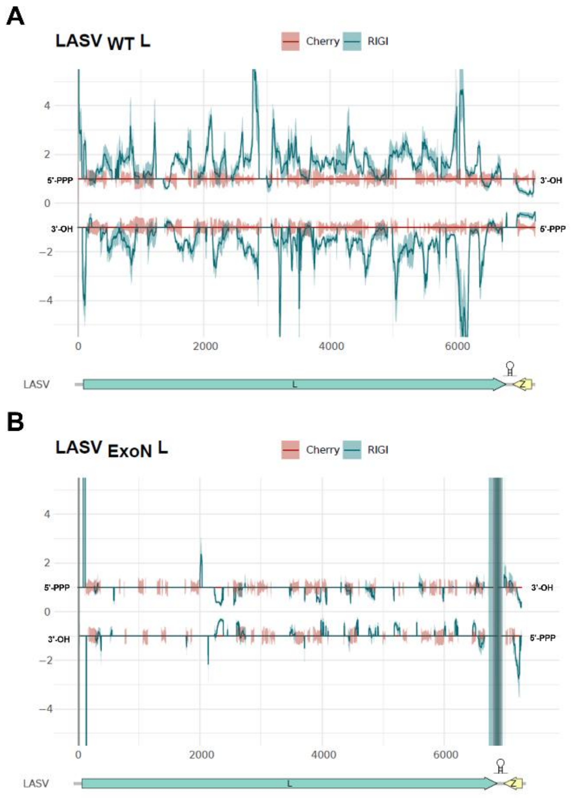

Figure S5. Enrichment of RIG-I-associated L segment regions during LASV infection.

The AP mCherry and RIG-I normalized coverages were standardized to the corresponding Total samples. The normalized RIG-I coverage was then compared to mCherry coverage (non-specific binding). The x-axis represents the position along the viral segment, and the y-axis shows the fold enrichment in AP between RIG-I compared to mCherry. Normalized RIG-I binding is plotted as positive values for the positive strand and as negative values for the negative strand. Data are presented for the L segment of $LASV_{WT}$ (A) and $LASV_{ExoN}$ (B). Data represent the mean of three independent biological replicates. The 5' and 3' ends of each genomic or antigenomic segment are indicated (5'-PPP or 3'-OH), as well as the localization of the hairpin structures in the intergenic regions.

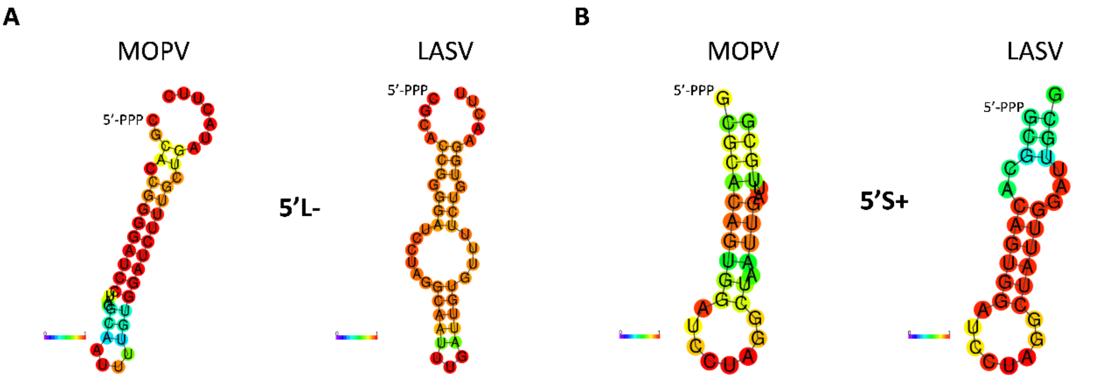

Figure S6. Visualization of the potential RNA fold of the immunogenic MOPV RNAs. RIG-I associated 5'L- (A) and 5'S+ (B) MOPV RNA sequences and the corresponding LASV RNA sequences were analyzed using RNA fold (http://rna.tbi.univie.ac.at/) and are represented as minimum free energy predictions. The color indicates the base-pair probabilities from 0 to 1.
